# A bacterial Goldilocks mechanism

**DOI:** 10.7554/eLife.54244

**Published:** 2020-01-24

**Authors:** Irene M Kim, Hendrik Szurmant

**Affiliations:** College of Osteopathic Medicine of the PacificWestern University of Health SciencesPomonaUnited States

**Keywords:** peptidoglycan, D,L-endopeptidase, two-component signaling, WalR, WalK, homeostasis, *B. subtilis*

## Abstract

*Bacillus subtilis* can measure the activity of the enzymes that remodel the cell wall to ensure that the levels of activity are ‘just right’.

**Related research article** Dobihal GS, Brunet YR, Flores-Kim J, Rudner DZ. 2019. Homeostatic control of cell wall hydrolysis by the WalRK two-component signaling pathway in *Bacillus subtilis*. *eLife* **8**:e52088. doi: 10.7554/eLife.52088

When mollusks get bigger, their shells grow with them to accommodate the changing shape and size of the organism being housed. Something similar also happens in bacteria. The cell wall of most bacteria consists of a single macromolecule called peptidoglycan that surrounds the cell and is made up of modified sugars that are crosslinked through peptide side chains. Like sea shells, bacteria come in different sizes and the cell wall dictates their shape (Cabeen and Jacobs-Wagner, 2007). The cell wall also protects bacteria from adverse environmental conditions, but it must be constantly remodeled so that rapid bacterial growth and division can take place. The enzymes in charge of this remodeling process are called autolysins, and their activity must be regulated to stop bacteria from losing their cell wall.

Signal transduction systems are protein systems that detect molecular or physical cues and translate them into an appropriate cellular response. In bacteria, signal transduction is commonly regulated by two-component systems, known as TCS for short (Zschiedrich et al., 2016). Usually the two components are a signal detector and a transcription factor that communicate with one another through the transfer of a phosphoryl group.

An important TCS in the soil bacterium *Bacillus subtilis* and other related bacteria is the WalRK system, which is essential for viability (Szurmant, 2012). The system, which is comprised of the signal-detecting protein WalK and the transcription factor WalR, gets its name from its role in maintaining the cell wall (Dubrac et al., 2007). Together these two components regulate the expression of several autolysin genes, including those for the enzymes LytE and CwlO, which are required for cell elongation (Salzberg et al., 2013). Now, in eLife, David Rudner and colleagues at Harvard Medical School – including Genevieve Dobihal and Yannick Brunet as joint first authors, along with Josué Flores-Kim – report on how the WalRK system in *B. subtilis* detects and responds to autolysin levels (Dobihal et al., 2019).

Dobihal et al. first observed that *B. subtilis* can measure the levels of autolysin activity and, if they are too low for the cell to grow, can adjust them accordingly. Next, they examined if the reverse is also true: can the cell identify if autolysin activity is too high to retain the protective shell, and reduce autolysin expression appropriately? Indeed, when the autolysin LytE is artificially overproduced, the cell reduces endogenous production of this enzyme. Thus the bacterium employs homeostatic control to ensure that autolysin activity is 'not too much, not too little, but just right', just like in the tale of Goldilocks and the three bears (Figure 1). This equilibrium is important given that mis-regulated autolysin activity can lead to cell lysis and defects in the permeability of the membrane.

**Figure 1.**
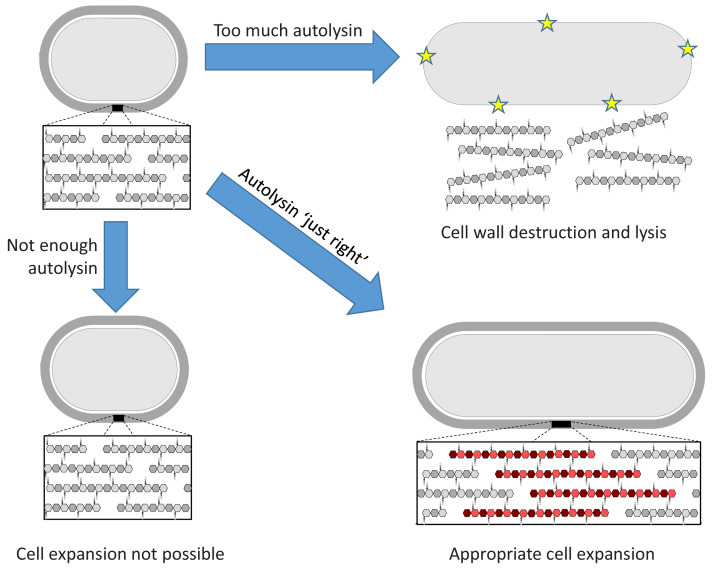
The Goldilocks principle applied to bacterial cell wall homeostasis. The bacterial cell wall (top left) consists of sugar strands (hexagons) that are crosslinked via peptide bonds between their peptide sidechains (small circles). Cell expansion requires the incorporation of new cell wall material. Autolysin enzymes cleave the peptide crosslinks to allow for expansion. Insufficient autolysin activity prevents expansion and thus growth (bottom left). Uncontrolled autolysin activity results in cell wall destruction and lysis (indicated by yellow stars, top right). When the autolysin activity is ‘just right’, the cell wall expands (red) and its integrity is maintained (bottom right).

Dobihal et al. then used several reporters to measure the expression of different genes regulated by WalR, and found they all responded similarly to the overexpression and deletion of the gene for LytE. This suggests that LytE and CwlO activity is directly detected by the WalRK system, but the precise signal used by the WalRK system to detect this activity remained unknown, as did the mechanism of detection.

WalK is a multi-domain membrane-spanning protein that has two domains commonly associated with signal detection: one of these domains faces the outside of the cell whereas the other faces the inside (Fukushima et al., 2011). WalK interacts with two other proteins that inhibit its activity, WalH and WalI (Szurmant et al., 2007; Szurmant et al., 2008). The signal for autolysin levels could be perceived by either of the two inhibitor proteins or by one of the signal detection domains of WalK. Dobihal et al. deleted domains in WalH, WalI and WalK to determine which protein detected the signal, demonstrating that the WalK domain that faces the outside of the cell is the only one required.

But what is the signal detected by WalK? LytE and CwlO are both able to cleave peptide bonds, probably to reduce crosslinks in the cell wall (Bisicchia et al., 2007). WalK could therefore be responding to a physical signal, such as a change in the tension exerted by a cell wall with too many or too few crosslinks. Alternatively, the signal could be of a chemical nature, such as a peptide being released when the autolysins remodel the cell wall. To distinguish between these two possibilities, Dobihal et al. exposed the purified cell wall of *B. subtilis* to the CwlO enzyme in vitro, and then applied the cleavage products of the reaction to *B. subtilis* cultures. The results showed that the cleavage products of CwlO can affect the expression of genes regulated by WalR. Exactly which molecule interacts with WalK to relay the signal remains unknown.

The findings by Dobihal et al. contribute to our understanding of the WalRK two-component system in *B. subtilis*. The spherical bacteria *Staphylococcus aureus* and *Streptococcus pneumoniae* are distant relatives of *B. subtilis* and also use the WalRK system to modulate autolysin gene expression, despite not growing by cell wall elongation (Ng and Winkler, 2004; Dubrac et al., 2007). Differences in domain architecture of WalK (*S. pneumoniae*) or cell wall crosslinks (*S. aureus*) dictate that the signal to modulate WalRK activity must be different from the one used by *B. subtilis*. Even in *B. subtilis* previous results suggested that there might be additional signals detected by WalK that are related to cell division (Fukushima et al., 2011). Thus, a unifying theme for the role of WalRK in all these bacteria remains unclear, and requires additional studies to build on these exciting new insights.
